# Phytate degradation in composite wheat/cassava/sorghum bread: Effects of phytase‐secreting yeasts and addition of yeast extracts

**DOI:** 10.1002/fsn3.3754

**Published:** 2023-10-13

**Authors:** Serafina Lídia Vilanculos, Ulf Svanberg, Thomas Andlid

**Affiliations:** ^1^ Departamento de Engenharia Química, Faculdade de Engenharia Universidade Eduardo Mondlane Maputo Mozambique; ^2^ Department of Life Sciences/Food and Nutrition Science Chalmers University of Technology Gothenburg Sweden

**Keywords:** composite flour bread, phytase, phytate, phytate degradation, *Pichia kudriavzevii* TY13, yeast extract

## Abstract

Iron deficiency anemia is highly prevalent in developing countries due to the consumption of cereal‐based foods rich in phytate that chelates minerals such as iron and zinc making them unavailable for absorption by humans. The aim of the present study was to degrade phytic acid in composite flour (wheat/cassava/sorghum) bread by the addition of phytase‐producing yeasts in the baking process to achieve a phytate‐to‐iron molar ratio <1 and a phytate‐to‐zinc molar ratio <15, ratios needed to achieve an enhanced absorption by humans. The high‐phytase (HP)‐producing yeasts were two *Saccharomyces cerevisiae* (YD80 and BY80) that have been genetically modified by a directed mutagenesis strategy, and *Pichia kudriavzevii* TY13 isolated from a Tanzanian lactic fermented maize gruel (*togwa*) and selected as naturally HP yeast. To further improve the phytase production by the yeasts, four different brands of phytase‐promoting yeast extracts were added in the baking process. In addition, two yeast varieties were preincubated for 1 h at 30°C to initiate phytase biosynthesis. The phytate content was measured by high‐performance ion chromatography (HPIC) and the mineral content by ion chromatography (HPIC). The results showed that all three HP yeasts improved the phytate degradation compared with the composite bread with no added HP yeast. The composite bread with preincubated *S. cerevisiae* BY80 or *P. kudriavzevii* TY13 plus Bacto yeast extract resulted in the lowest phytate content (0.08 μmol/g), which means a 99% reduction compared with the phytate content in the composite flour. With added yeast extracts from three of the four yeast extract brands in the baking process, all composite breads had a phytate reduction after 2‐h fermentation corresponding to a phytate: iron molar ratio between 1.0 and 0.3 and a phytate: zinc molar ratio <3 suggesting a much‐enhanced bioavailability of these minerals.

## Graphical abstract

Composite breads with *P. kudriavzevii* TY13 and addition of yeast extracts from three of the four yeast extract brands in the baking process had a phytate reduction after 2‐h fermentation time to obtain a phytate: iron molar ratio between 1.0 and 0.3 and a phytate: zinc molar ratio <3 that suggests an enhanced bioavailability of these minerals.

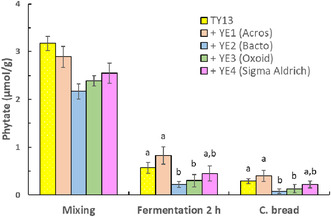

## 1 INTRODUCTION

Wheat bread is an important staple food in Mozambique, while the country does not produce wheat flour and therefore needs to import wheat at high cost. Mozambique produces other cereals and tubers that can be used as substitutes for wheat flour in bread making (Eduardo et al., 2014). However, whole cereal flours are rich in phytate (*myo*‐inositol hexaphosphate) (Garcia‐Estepa et al., 1999; Kumar et al., 2010), an antinutrient that strongly chelates minerals such as iron and zinc making them unavailable for absorption by humans (Brune et al., 1992; Hurrel, 2004). Diets based on cereals and legumes are, therefore, associated with high prevalence of iron deficiency anemia in children and women in low‐ and middle‐income countries (Hurrell et al., 2002; Tatala et al., 1998; Zimmermann et al., 2005).

To increase iron and zinc bioavailability, phytate needs to be degraded by phytase, an enzyme widespread in plants (Steiner et al., 2007) and in certain microorganisms (Hellström et al., 2010; Howson & Davis, 1983; Nakamura et al., 2000; Olstorpe et al., 2009; Sandberg & Andlid, 2002). Phytases belong to a subgroup of acid phosphatases which catalyze the stepwise hydrolysis of phytate into lower phosphate esters of *myo*‐inositol, thereby releasing soluble inorganic phosphate and nonchelated minerals, which become available for human intestinal absorption (Konietzny & Greiner, 2002). To improve iron absorption, the phytate content needs to be degraded to a phytate: iron molar ratio <1 and preferably <0.4 (Hurrel, 2004) which for cereals corresponds to a reduction higher than about 98%, and for an enhanced zinc absorption the phytate: zinc molar ratio needs to be <15 (Nävert et al., 1985). To achieve such high phytate degradation in breadmaking, suitable conditions to activate the cereal phytase must be obtained at the dough preparation stage, that is, optimal pH and temperature. Attempts to adjust the pH in preparation of wholemeal wheat doughs with organic acids and fermenting for 2 h at 37°C resulted in a 96% degradation of phytate (Türk et al., 1996), and prolonging the incubation time (4 h at 30°C) resulted in an almost complete phytate degradation in wholemeal wheat flour doughs (Fretzdorff & Brummer, 1992). Presoaking of sorghum flour for 3 h at room temperature and optimal pH conditions resulted in a 90% degradation of the phytate content in composite wheat/cassava/sorghum bread (Vilanculos & Svanberg, 2021). On the other hand, certain flours and conditions may be sufficient, for example, rye has high intrinsic phytase activity which may lead to complete degradation of phytate in commercial rye bread (Egli et al., 2003; Nielsen et al., 2007).

Several researchers have also explored the combined effect of intrinsic and exogenous phytase on phytate degradation. Türk et al. (1996) observed a synergistic effect of the phytases from flour and yeast in baking of wholemeal wheat bread. Haros et al. (2001) added an exogenous microbial phytase from *Aspergillus niger* in baking of wholemeal wheat bread and reported an increased phytate degradation from about 60% to 90% and a similar effect of added phytase from *Aspergillus niger* was reported by Rosell et al. (2009). However, since no commercial food‐grade phytase is available, it would be an advantage if the phytase could be administered via the yeast in the baking process. One such nonconventional yeast, *Pichia kudriavzevii* TY13, with a high capacity to synthesize and release phytase to the surrounding medium was recently isolated from a Tanzanian lactic acid fermented maize gruel (*togwa*) (Hellström et al., 2010, 2015). The species *P. kudriavzevii* is ubiquitous in nature and very common in traditional fermented foods in all parts of the world (Yunfei et al., 2023). It is currently being explored for its potential in food production, as probiotic and in biotechnology, for instance, known to contribute to a pleasant aroma profile in, for example, wine and sourdough bread (Dan et al., 2020). Strains within a species do, however, differ, and TY13 was selected among other yeasts, including other strains of *P. kudriavzevii*, as being superior in phytate degradation. An important characteristic of the new yeast strain TY13 is that presence of inorganic phosphate does not inhibit the synthesis and release of phytase (Hellström et al., 2012). We have recently shown that an increased phytate degradation can be obtained in composite wheat/cassava/sorghum bread when *P. kudriavzevii* TY13 and growth‐promoting yeast extract were included in the baking process (Vilanculos et al., 2022).

There are several commercial products of so‐called nutritional yeast, which is dried food‐grade yeast extract in the form of flakes or powder. These are commonly used as nutritional supplement (and flavor), for richness in fiber (the yeast cell wall; glucans mannans), B vitamins, proteins, amino acids, minerals, and other micronutrients (Vieira et al., 2016). We, therefore, propose that the benefit of including yeast extract as an ingredient in bread may be two‐fold: (i) direct nutritional by the extract itself and (ii) increased yeast phytase production by live yeast *P. kudriavzevii* TY13 resulting in more complete phytate degradation leading to improved mineral availability of the final bread. The composition of commercial yeast extracts, however, depends on the history of the yeast, for example, originating from brewing, fermentation medium, yeast strain, process of autolysis, and extraction method, and can therefore vary between manufacturers and even between lots of the same brand.

In the present work, we, therefore, ask whether different types of yeast extract in combination with *P. kudriavzevii* TY13 will lead to different degrees of phytate degradation in composite bread based on wheat, wholemeal sorghum, and cassava flours. The ultimate goal is to obtain enough phytate reduction in the composite bread to achieve an improved absorption of iron and zinc by humans. Two strains of genetically modified *S. cerevisiae* strains (BY80 and YD80) with increased capacity to synthesize phytase (Veide & Andlid, 2006) were for comparison also included in the baking process.

## 2 MATERIALS AND METHODS

### 2.1 Flours for breadmaking

The ingredients used for the composite flour were wheat (*Triticum aestivum*) flour with an extraction rate of 72% (Frebago 1050 Bagerivetemjöl, Sweden), cassava (*Manihot esculenta* Crantz) flour, and nontannin white whole sorghum (*Sorghum bicolor*) flour from Inhambane province in Mozambique.

The cassava roots were peeled, washed, cut into pieces, and sun dried for 4 days. During the drying period, the cassava pieces were flipped three times per day to prevent from mold growth and then milled, packed, and stored. The sorghum grains were harvested, and then washed and damaged grains were sorted out. After sun drying for 2 days, the grains were milled at 100% extraction rate, and finally packed and stored.

### 2.2 Preparation of yeast culture

Three high‐phytase yeasts were used: *P. kudriavzevii* TY13 and genetically modified *S. cerevisiae* strains BY80 and YD80. These were long‐term stored in 15% glycerol solution at −80°C and short‐term stored during experimental periods on YPD agar plates at +4°C (10 g/L yeast extract, 20 g/L peptone, 20 g/L D‐glucose, and 20 g/L agar). As precultures, 5 mL YPD in Falcon tubes were inoculated with yeast culture from fresh YPD agar plates and incubated in a rotating carousel for 24 h at 30°C. The precultures were inoculated into yeast biomass production flasks: a set of 250 mL shake flasks containing 200 mL YPD which were incubated for 24 h at 30°C under rotary shaking. To collect the yeast biomass, cultures were centrifuged at 4500 ***g*** using a Heraeus Multifuge (Kendro, Osterode, Germany) for 10 min, the supernatants were discarded, and the compressed yeast pellets were stored in cold room (+4°C) until used within a few days.

The *S. cerevisiae* strains BY80 and YD80 were in our previous work transformed into high‐phytase‐producing strains by modifications in the *PHO* system. The YD80 was transformed by deletion of the negative regulator *PHO80* in combination with overexpression of *PHO5* and the BY80 strain was transformed with overexpression of the transcriptional activator *PHO4* (Veide & Andlid, 2006).

### 2.3 Bread‐making procedure

Bread was prepared by mixing 100 g of wheat flour, 50 g of cassava flour, and 50 g of whole sorghum flour in a dry stage for 1 min in a kitchen aid (Artisan, Model 5KSM 150, USA), and then 135 mL of water and the rest of the ingredients were added, sugar 4 g, salt 2 g, baker's yeast 4 g (ordinary commercial *Saccharomyces cerevisiae*; Swedish yeast company), margarine 6 g, and ascorbic acid 0.1 g. The mixture was then blended for 2 min at speed level 2 and 3 min at speed level 4. At the mixing stage, either 2 g of compressed *P. kudriavzevii* TY13 or one of the two genetically modified *S. cerevisiae* yeast strains (YD80 and BY80) was added, and the pH of the dough was adjusted to 4.0 by adding a 20% lactic acid solution (~20 mL). To improve the growth of *P. kudriavzevii* TY13, 2.0 g of four different brands of yeast extract (see Table 2) was added at the mixing stage. Preincubation of *S. cerevisiae* YD80 and BY80 was done in 100 mL of water at 30°C adjusted to pH 4.0 with lactic acid and addition of 3 g of sucrose, and then the mixture was kept in a heating cabinet at 30°C for 1 h. The incubated yeast mixture was then added to the dry bread ingredients with an additional 35 mL of water. The dough was then covered with cotton cloth and left to ferment for 1 h at room temperature (~21°C). After weighing, the dough was then divided into four round‐shaped 80‐g pieces and placed into baking pans to be baked off or to ferment for another 1 h. The rolls were baked for 8 min at 250°C in a kitchen oven. Each recipe was baked in triplicate.

During the baking process, samples were taken at all stages; after mixing, after 1 and 2 h of fermentation, and at 30 min after baking and cooling. The withdrawn samples were weighed and placed into a plastic test tube and immediately frozen at −20°C and then lyophilized for 3 days.

### 2.4 Phytate extraction and analysis

The phytate content measured as IP6 was analyzed on an HPLC system as described in detail by Carlsson et al. (2001) with minor modifications by Vilanculos and Svanberg (2021). In brief, the chromatograph consisted of an HPLC pump (Waters model 626) equipped with an Omni Pac PAX‐100 (4 mm × 250 mm) analytical column and a PAX‐100 (4 mm × 50 mm) guard‐column (Dionex Corp., Sunnyvale, CA, USA). The IP_6_ was detected and quantified after a postcolumn reaction with Fe(NO_3_)_3_x9H_2_O and the absorbance was measured at 290 nm using an ultraviolet detector.

### 2.5 Mineral extraction and analysis

The content of iron and zinc was determined using an ion chromatography system coupled with postcolumn derivatization and ultraviolet–visible detection at 500 nm according to the method by Fredrikson et al. (2002), with minor modifications as described by Vilanculos and Svanberg (2021).

### 2.6 Phosphate analysis

The phosphate content in the yeast extracts and the composite flour components was determined according to the HPLC method described by Qvirist et al. (2015) with minor modifications according to Vilanculos et al. (2022).

### 2.7 Determination of dry matter

The dry matter content was determined by using a balance device 310M and a HA300 dryer (Precisa Dietikon, Switzerland). Approximately 0.5 g of food material was weighed into the device and heated to a temperature of 80°C under reduced pressure (900 mbar) until constant weight.

### 2.8 Determination of pH

The pH was measured using Mettler Toledo MA 235 pH/Ion Analyzer. A 16‐g dough piece was weighed and put into a flask tube and 8 g of water was added, then stirred using an electromagnetic plate (Retsch, Rühra mag type Mo12**)** and stirrer bar for 5 min. Finally, the pH was read using the pH‐meter probe.

### 2.9 Determination of total N and free amino acids

The total nitrogen content of yeast extracts was determined by the Dumas combustion method using a TruMac nitrogen analyzer (LECO, St Joseph, MI, USA). The combustion process converts covalently bound nitrogen into nitrogen gas (N_2_) that is quantified with the use of a conductivity cell. The content of free amino acids was analyzed according to Hinchcliffe et al. (2019). Yeast samples (10 mg) were extracted by using diluted HCl. Separation and quantification of the amino acids was performed using Agilent 1260–1290 infinity LC System on a thermos Luna column C18(2) (250 × 4.6 mm and particle size 3 μm) at a flow rate of 0.7 mL/min. The Thermo‐Scientific Pierce Amino Acid Standard (20088) was used for quantification.

### 2.10 Chemicals

The used chemicals were hydrochloric acid, nitric acid, and lactic acid from Scharlau (Scharlab S.L., Spain), and iron nitrate, ascorbic acid, and 70% ethanol (Sigma Aldrich, Stockholm, Sweden). The deionized water was generated by Millipore Milli‐Q plus ultrapure water system (Millipore, Solna, Sweden). The four yeast extracts (YE1 to YE4) used for the baking studies were as follows: YE1 (Acros organics from Fisher Scientific, UK), YE2 (Bacto yeast extract from Difco Laboratories, UK), YE3 (Oxoid yeast extract from Oxoid Ltd, UK), and YE4 (yeast extract from Sigma Aldrich, Stockholm, Sweden).

### 2.11 Statistical analysis

Data are presented as mean values ± standard deviation of at least three baking replicates analyzed in duplicates. All statistical analyses were performed using SPSS (version 15.0, SPSS Inc., Chicago, IL) software. Mean values were compared by analysis of variance, and determination of significant differences between variables was made with Tukey's HSD posthoc multiple range test. Differences were considered to be significant at *p* < .05.

## 3 RESULTS

### 3.1 Phytate and minerals from the ingredient flours of composite bread

Table 1 shows that the whole sorghum flour had the highest content of phytate, iron, and zinc, respectively, 11.8 μmol/g, 37.3 μg/g, and 16.0 μg/g. The cassava flour had the lowest content of the same compounds, respectively, 4.0 μmol/g, 6.4 μg/g, and 4.0 μg/g. Iron and zinc content in the composite bread was then 14.8 and 6.5 μg/g, respectively. The amount of inorganic phosphate in the three composite flours was for wheat 1.59 mg/g, sorghum 1.32 mg/g, and cassava 1.23 mg/g. The highest phytate‐to‐iron molar ratio of 36 was obtained in the cassava flour while whole sorghum flour had the lowest ratio of 18. The molar ratio of phytate to zinc was generally higher with a value of 70 for wheat flour and 48 for whole sorghum flour. By mixing wheat, sorghum, and cassava in proportion 2:1:1, the resulting composite flour had phytate molar ratios of 20 and 60 for iron and zinc, respectively.

**TABLE 1:** Phytate, phosphate, and minerals content per gram dry weight of the ingredient flours for composite bread.

Type of flour	Phytate (μmol/g)	Fe (μg/g)	Phytate: Iron molar ratio	Zn (μg/g)	Phytate: Zinc molar ratio	Phosphate (mg/g)
Whole sorghum	11.8 ± 0.2	37.3 ± 0.9	17.8	16.0 ± 0.5	48.2	1.32 ± 0.06
Wheat	4.4 ± 0.2	11.0 ± 0.5	21.8	4.0 ± 0.4	70.3	1.59 ± 0.02
Cassava	4.0 ± 0.1	6.4 ± 0.4	36.2	4.0 ± 0.3	65.2	1.23 ± 0.03
Composite flour	5.6 ± 0.1	15.6 ± 0.4	19.9	6.1 ± 0.2	59.9	1.46 ± 0.07
Composite bread^a^	1.15–0.08	14.8 ± 0.8^b^	4.3–0.3	6.5 ± 0.6^b^	11.6–0.8	n.d.

^a^ Highest and lowest content of phytate in the composite breads.

^b^ Average and S.D. of *n* = 4.

### 3.2 Phytate degradation in composite bread with added high‐phytase yeasts

**FIGURE 1:**
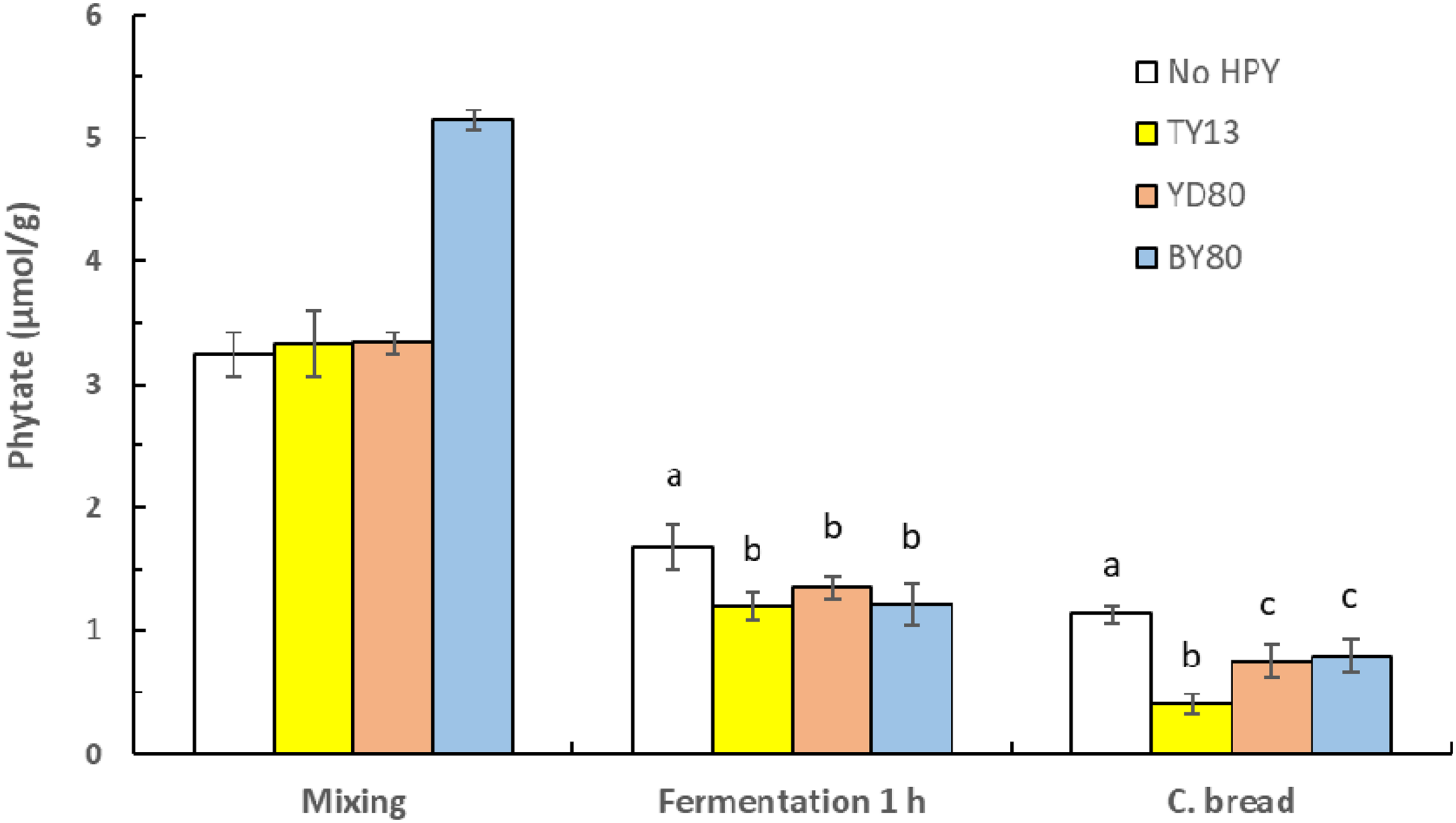
Phytate content during baking of composite bread, after mixing, fermentation for 1 h at ambient temperature and in the composite bread with addition of either *P. kudriavzevii* TY13, *S. cerevisiae* YD80 or BY80. Samples within each treatment showing a different letter (a–c) are significantly different *p* < .05 (No HPY = no addition of high‐phytase yeast).

The capacity of our strain TY13 to be catabolically active in the prevailing conditions (utilizing available energy and nutrients) and to use the liberated energy and building blocks to synthesize the enzyme phytase was demonstrated in our study. Figure 1 shows that after fermentation for 1 h at room temperature, the doughs with added *P. kudriavzevii* TY13 and BY80 and YD80 (all high‐phytase yeasts) had a similar phytate content, ~1.25 μmol/g. In comparison with the initial phytate content in the composite flour, that means an almost 80% reduction. In the composite breads, the phytate content was further reduced with the lowest value of 0.41 μmol/g in bread with added *P. kudriavzevii* TY13 that was significantly lower (*p* < .05) than in the composite breads with added BY80 and YD80, 0.75 μmol/g. In the bread without addition of high‐phytase yeasts (yet containing commercial Baker's yeast), the phytate content was significantly higher, 1.13 μmol/g.

**FIGURE 2:**
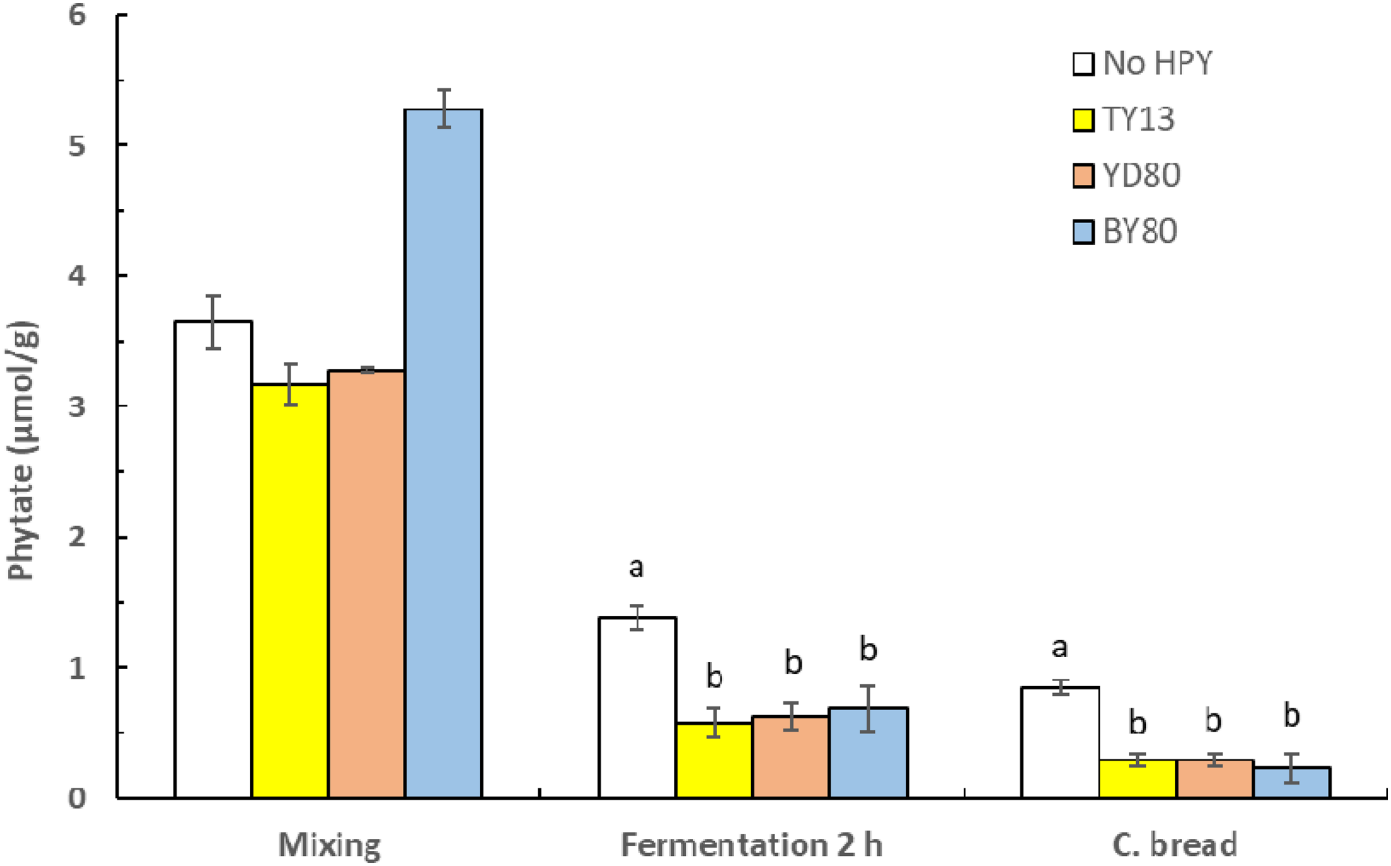
Phytate content during baking of composite bread, after mixing, fermentation for 2 h at ambient temperature and in the composite bread with addition of either *P. kudriavzevii* TY13, *S. cerevisiae* YD80 or BY80. Samples within each treatment showing a different letter (a, b) are significantly different *p* < .05. (No HPY = no addition of high‐phytase yeast).

Figure 2 shows that after 2‐h fermentation, the phytate content was further reduced in the composite breads with high‐phytase yeasts, to levels of about 0.26 μmol/g, significantly lower (*p* < .05) than in the bread without phytase‐producing yeast (0.85 μmol/g).

### 3.3 The effect of different yeast extracts on phytate degradation

The amount of added yeast extract (YE) was based on YPD, which is a standard complex yeast medium containing 1% YE (wt/vol). We also used 1% YE in our prior work, where we initially found a phytase‐inducing effect by YE in TY13 (Hellström et al., 2015). However, in the present study, we reduced the YE concentration to 0.5% since preliminary experiments showed no difference between 1% and 0.5%.

**FIGURE 3:**
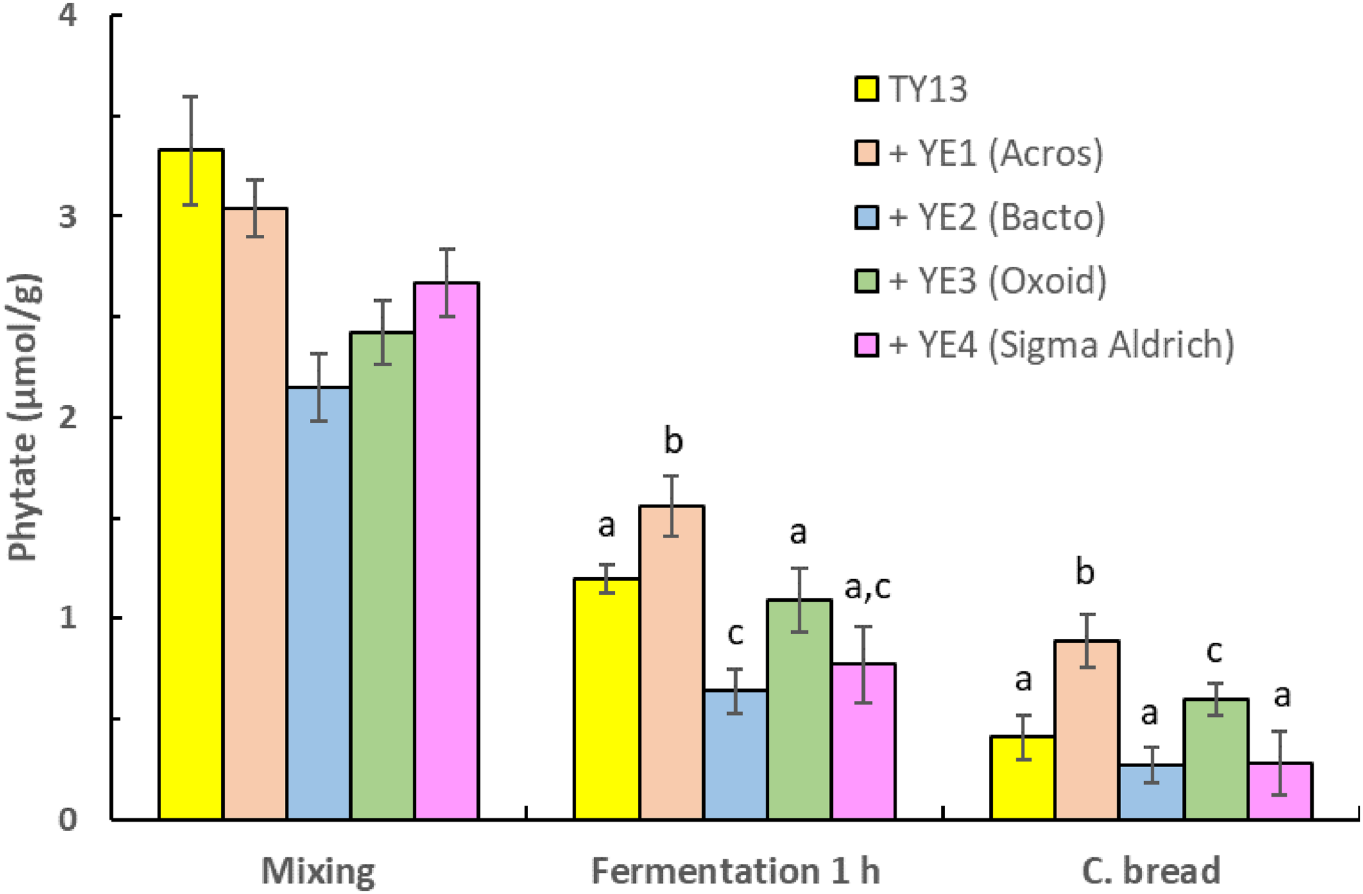
Phytate content during baking of composite bread with addition of *P. kudriavzevii* TY13 and four different types of yeast extracts (2.0 g) at mixing stage and with 1‐h fermentation at ambient temperature. Samples within each treatment showing a different letter (a–c) are significantly different *p* < .05.

**FIGURE 4:**
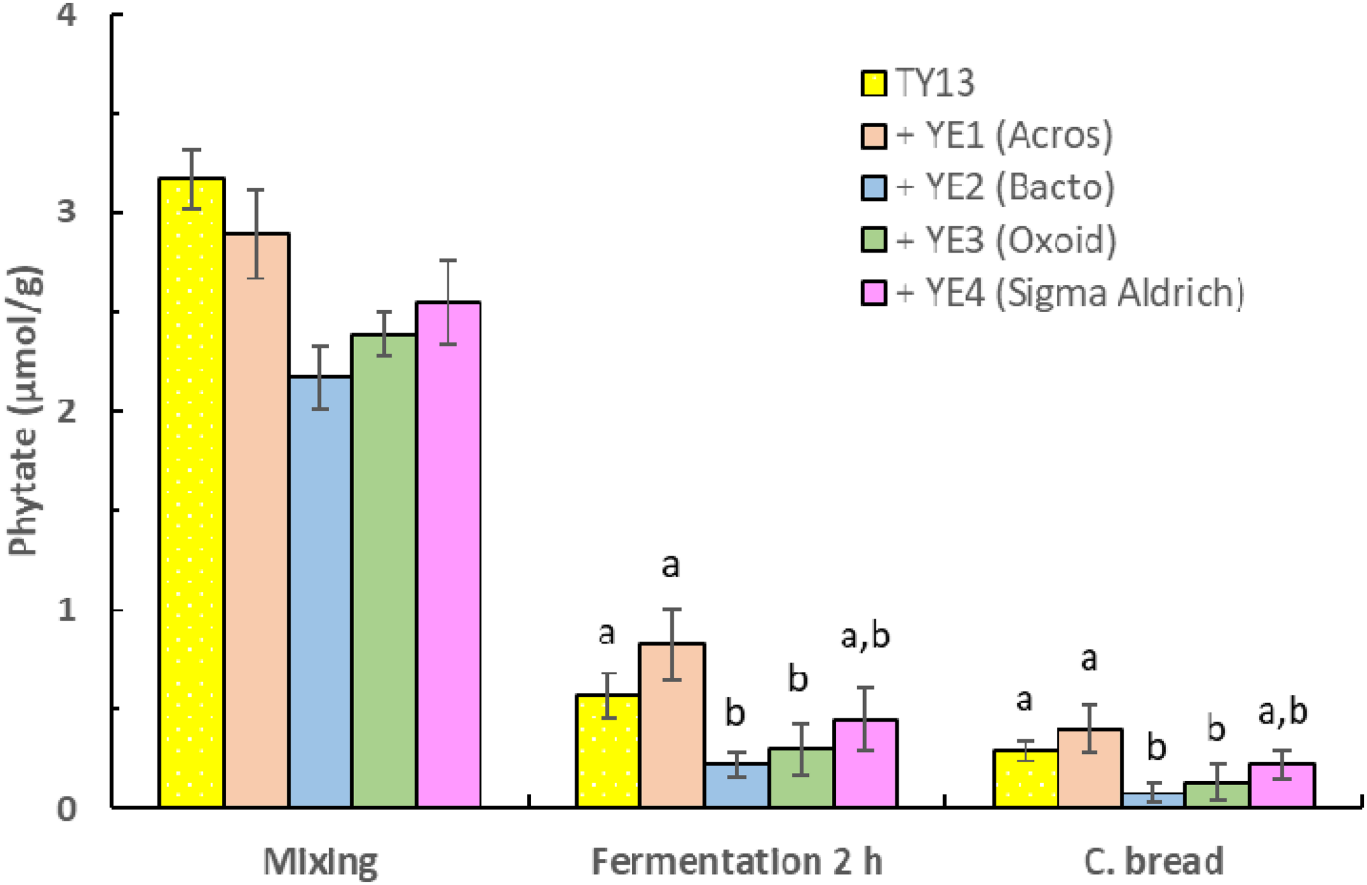
Phytate content during baking of composite bread with addition of *P. kudriavzevii* TY13 and four different types of yeast extracts (2.0 g) at mixing stage and with 2‐h fermentation at ambient temperature. Samples within each treatment showing a different letter (a, b) are significantly different *p* < .05.

The four different types of yeast extracts (YE1‐4) used in this study had significantly different compositions of phosphate, total nitrogen, and free amino acid content. Table 2 shows that YE1 (Acros Organics), YE2 (Bacto), YE3 (Oxoid), and YE4 (Sigma Aldrich) had a phosphate content ranging between 25.8 and 30.9 mg/g, a total sum of free amino acids ranging from 35.5 and 37.3 g/100 g, and with total of nitrogen of about 11 g/100 g. These yeast extracts were added to the dough during the mixing stage to induce a higher metabolic activity of *P. kudriavzevii* TY13 and thereby an increased synthesis of phytase. Figures 3 and 4 show that the phytase degrading effect of *P. kudriavzevii* TY13 was differently affected by the four yeast extract varieties. After 1‐h fermentation, the composite bread with added YE1 had a significantly higher (*p* < .05) phytate content (0.89 μmol/g) when compared with the bread without yeast extract (0.41 μmol/g) but after 2‐h fermentation, the phytate degradation was about the same in the two composite breads (~0.35 μmol/g). The addition of the three other yeast extracts at the mixing stage resulted in a significantly lower phytate content (*p* < .05), between 0.08 and 0.22 μmol/g in the composite bread after 2‐h fermentation compared with the bread without added yeast extract (Figure 4).

**TABLE 2:** Phosphate and nitrogen composition of yeast extract batches.^a^

Component	YE1	YE2	YE3	YE4
Phosphate (mg/g)	29.7 ± 0.7	25.8 ± 0.6	27.6 ± 0.3	30.9 ± 1.1
Total nitrogen (g/100 g)	11.2 ± 0.01	11.1 ± 0.04	11.0 ± 0.02	11.6 ± 0.04
*Amino acids (g/100 g)*				
Gly	0.90 ± 0.04	1.06 ± 0.03	1.0 ± 0.03	0.96 ± 0.02
Ala	3.41 ± 0.09	2.66 ± 0.08	2.51 ± 0.06	2.83 ± 0.13
Ser	1.77 ± 0.07	2.02 ± 0.05	1.87 ± 0.06	1.87 ± 0.05
Pro	1.84 ± 0.09	1.88 ± 0.04	1.62 ± 0.11	1.78 ± 0.09
Val	2.00 ± 0.08	2.32 ± 0.06	2.34 ± 0.05	2.13 ± 0.04
Thr	1.25 ± 0.04	1.52 ± 0.05	1.47 ± 0.03	1.39 ± 0.04
Ile	1.75 ± 0.05	2.06 ± 0.05	2.00 ± 0.03	1.82 ± 0.03
Leu	2.36 ± 0.08	3.01 ± 0.09	3.06 ± 0.05	2.85 ± 0.06
Asp	2.32 ± 0.12	2.19 ± 0.05	2.01 ± 0.14	2.10 ± 0.12
Lys	2.30 ± 0.11	2.43 ± 0.06	2.17 ± 0.12	2.32 ± 0.07
Glu	3.14 ± 0.10	3.67 ± 0.14	4.02 ± 0.09	3.53 ± 0.12
Met	1.52 ± 0.07	1.50 ± 0.04	1.42 ± 0.07	1.41 ± 0.06
His	1.78 ± 0.09	1.68 ± 0.04	1.53 ± 0.12	1.60 ± 0.10
Phe	2.17 ± 0.07	2.34 ± 0.06	2.24 ± 0.08	2.19 ± 0.04
Arg	3.26 ± 0.16	3.17 ± 0.09	2.93 ± 0.19	3.05 ± 0.13
Tyr	3.31 ± 0.16	2.81 ± 0.07	2.57 ± 0.20	2.73 ± 0.17
Cys	1.06 ± 0.06	0.99 ± 0.08	0.89 ± 0.09	0.95 ± 0.06
Total Sum	36.4 ± 0.09	37.3 ± 0.06	35.7 ± 0.09	35.5 ± 0.10

^a^ YE1 = Acros, YE2 = Bacto, YE3 = Oxoid, YE4 = Sigma Aldrich.

### 3.4 The effect of yeast preincubation

**FIGURE 5:**
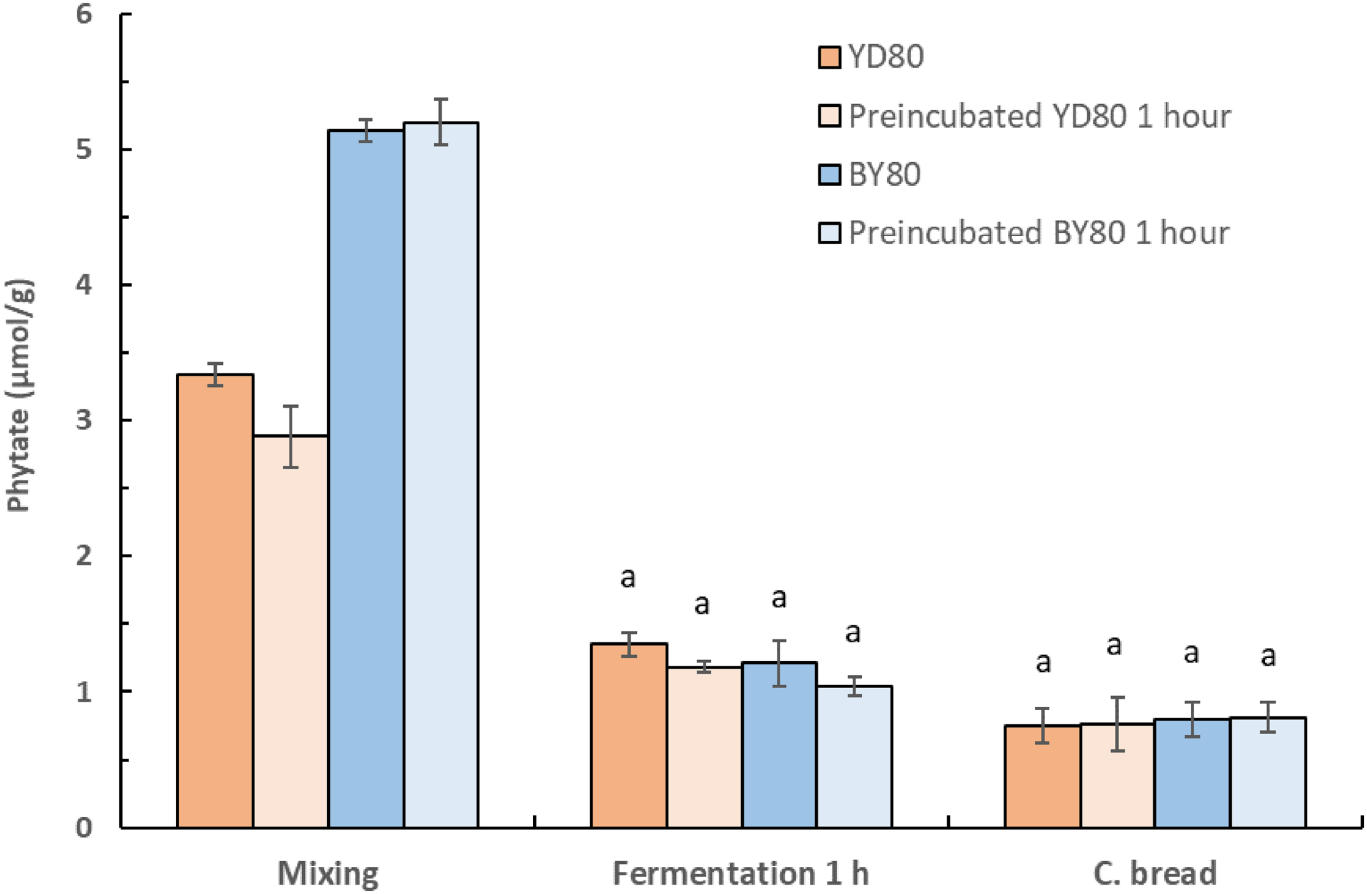
Phytate content during baking of composite bread with addition of *S. cerevisiae* YD80 or BY80, with and without preincubation for 1 h at 30°C, and fermentation for 1 h at ambient temperature. Samples within each treatment showing the same letter (a) are not significantly different *p* < .05.

**FIGURE 6:**
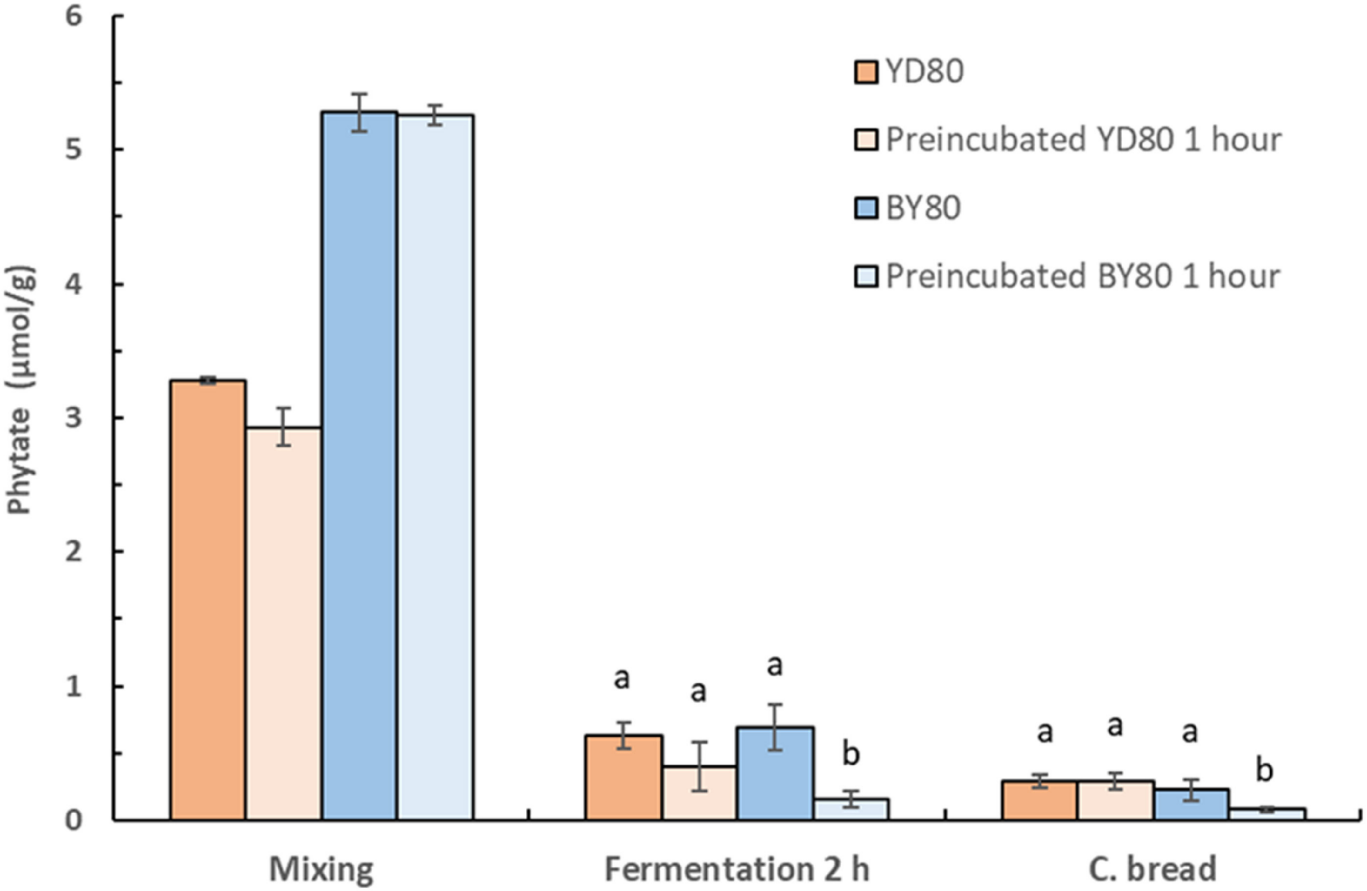
Phytate content during baking of composite bread with addition of *S. cerevisiae* YD80 or BY80, with and without preincubation for 1 h at 30°C, and fermentation for 2 h at ambient temperature. Samples within each treatment showing a different letter (a, b) are significantly different *p* < .05.

Figure 5 shows that the phytate content, in the composite breads with the modified varieties *S. cerevisiae* YD80 or BY80 fermented for 1 h, preincubated or not, was between 0.75 and 0.81 μmol/g and not significantly different from each other. However, after 2‐h fermentation (Figure 6), the lowest phytate content of 0.08 μmol/g was obtained in composite breads with added preincubated *S. cerevisiae* BY80 that was significantly lower than in the composite bread with added *S. cerevisiae* YD80, preincubated or not, with a phytate content of about 0.29 μmol/g.

## 4 DISCUSSION

### 4.1 Phytate and minerals from the ingredient flours of composite bread

Due to the inclusion of the bran fraction in the whole sorghum flour, the phytate content was high (11.8 μmol/g). As phytate in plant seeds is the main storage of phosphate and chelated minerals, it follows that high phytate coincides with high mineral content. The sorghum flour in our study contained more than three times higher iron content as compared with the wheat and cassava flours, because of a low extraction rate for the wheat flour (not fortified) and noninclusion of peels for the cassava roots during flour preparation. Earlier studies have reported similar values for phytate content in wheat flour (4.1 μmol/g) and cassava flour (2.9 μmol/g) by Lazarte et al. (2015) and for nontannin sorghum flour (12.4 μmol/g) by Svanberg et al. (1993). With a phytate content in the resulting composite flour (5.6 μmol/g), it means that the molar ratio of phytate to iron is higher than one which implies a low iron bioavailability (Hurrell et al., 2002), and a zinc molar ratio higher than 15 suggesting a low zinc absorption from the composite flour (Turnlund et al., 1984; Nävert et al., 1985) (see Table 1).

### 4.2 Phytate degradation with added *P. kudriavzevii* TY13, *S. cerevisiae* BY80 or YD80

The three added phytase synthesizing yeasts improved the phytate degradation compared with the composite bread with no added yeast. After 1‐h fermentation, the composite bread with added *P. kudriavzevii* TY13 had a significantly lower phytate content compared with the composite breads with added BY80 and YD80. An explanation could be that the *P. kudriavzevii* TY13 had a faster production and/or release of phytase into the dough, probably explained by its high capacity to synthesize phytase in the presence of inorganic phosphate (Hellström et al., 2012).

After 2‐h fermentation, all the composite breads with added high‐phytase yeasts had a reduced phytate content by about 95% (see Figure 2). The phytate‐to‐iron molar ratio was now close to 1.0 and the phytate‐to‐zinc molar ratio had been reduced to about 3, low enough to suggest an improved zinc bioavailability. However, in the composite breads with no addition of high‐phytase yeasts about 85% of the phytate content had been degraded, mainly as a result of activated endogenous phytase in the cereal flours at the mixing and fermentation stage at optimal pH conditions (Porres et al., 2001; Vilanculos & Svanberg, 2021).

### 4.3 The effect of different yeast extracts on phytate degradation

Yeast extracts are rich in aroma compounds and hence much used commercially as flavoring agents in many kinds of food. In addition, the yeast species *P. kudriavzevii* is also explored for natural production of aroma compounds (Dan et al., 2020; Yunfei et al., 2023), for instance, in sourdough bread together with *Lactobacillus plantarum*. Our focus was on nutritional enhancement and not on sensory properties. However, adding yeast extract and our nonconventional yeast TY13 has potential to also become sensorially pleasant.

The impact from adding yeast extract on phytate degradation was dependent on type/brand of yeast extract. Yeast extracts are nutritionally rich and complex with a nutrient composition dependent on factors including strain used (commonly from brewing or baking) and production process (autolysis, acid hydrolysis, or enzymes). The consequence is that yeast extracts may induce or inhibit phytase biosynthesis by live yeasts differently depending on brand used.

This has also been observed by Sørensen and Sondergaard (2014) who found that the nutrient composition of yeast extract brands, for example, nitrogen and carbon sources differently influenced the production of secondary metabolites like phytase. Raman et al. (2019), for example, reported that among tested nitrogen sources (yeast extract, meat extract, and peptone from casein) in cultivation of lactic acid bacteria, the highest cell concentration and phytase biosynthesis were achieved in yeast extract cultivation.

In our study, addition of yeast extract YE2 (Bacto) induced the highest phytate degradation of 99% in the composite bread after 2‐h fermentation that could be explained by a relatively higher amount of free amino acids (37.3 g/100 g) in combination with significantly lower phosphate content (2.58 g/100 g) in this yeast extract variety (see Table 2). However, it cannot be excluded that differences in micronutrients between the extracts, such as minerals and growth factors (e.g., vitamins, nucleotides, sterols), also influenced the phytase production.

High phosphate conditions are known to repress the synthesis of acid phosphatases (of which, e.g., *S. cerevisiae* phytases belong to) and phytases (Andlid et al., 2004), while low phosphate conditions result in their expression. A sharp decline in phytase production of *A. niger* was observed even at an inorganic phosphate concentration of 5 mM in the growth medium with no phytase production at 10 mM and above (Vats & Banerjee, 2002). In our study, the free inorganic phosphate concentration in the composite dough could be estimated to about ~23 mM, and with addition of yeast extract to about 28 mM (see Tables 1 and 2). However, the yeast variety *P. kudriavzevii* TY13 has previously been shown to produce extracellular phytase in the presence of inorganic phosphate up to 5 mM in lactic fermented Tanzanian maize gruels (togwa) (Hellström et al., 2012), and even shown some activity in synthetic medium with as high as 25 mM (Qvirist et al., 2016). Our findings, thus, show that *P. kudriavzevii* TY13 can exhibit significant phytase activity at a similar high phosphate concentration present in composite flour dough. In addition, presoaking of the sorghum ingredient with addition of *P. kudriavzevii* TY13 at a phosphate concentration of about 9 mM resulted in a phytate degradation in the composite bread similar to the results obtained in this study with addition of yeast extract at the mixing stage (Vilanculos et al., 2020, 2022).

### 4.4 The effect of yeast preincubation

Preincubation of *S. cerevisiae* YD80 and BY80 had no additional effect on the phytate degradation in the composite breads fermented for 1 h. However, after 2 h of fermentation with preincubated *S. cerevisiae* BY80, the phytate content in the composite breads was almost completely degraded, ~99%, while the phytate degradation in the composite bread with added preincubated *S. cerevisiae* YD80 was 94%. Both YD80 and BY80 were constructed to become constitutive high‐phytase producers (Veide & Andlid, 2006) at high inorganic phosphate concentration (~39 mM) which is higher than the inorganic phosphate concentration in the composite flour dough (23 mM). Preincubation with addition of sucrose for 1 h at optimal temperature for yeast growth and phytase activity obviously had a larger effect on BY80, resulting in a significantly higher phytate degradation (*p* < .05). An explanation could be that the variety BY80 has been shown to have a faster biomass production than YD80 under optimal growth conditions (Veide & Andlid, 2006). The composite breads with *S. cerevisiae* BY80 then had a phytate‐to‐iron molar ratio of 0.3 that indicates an improved iron bioavailability (Hurrel, 2004).

## 5 CONCLUSION

All three phytase‐secreting yeast varieties improved phytate degradation when added to baking of composite wheat/cassava/sorghum breads. However, only the natural isolate *P. kudriavzevii* TY13 is a non‐GMO yeast, whereas the other two have been genetically modified. The lowest phytate content (0.08 μmol/g) was obtained in the breads with addition of preincubated *S. cerevisiae* BY80 and *P. kudriavzevii* TY13 with additional yeast extract at the mixing stage. The phytate‐to‐iron molar ratio was then as low as 0.3 and the phytate: zinc molar ratio was <3 in the two composite breads strongly suggesting an enhanced mineral absorption by humans. Finally, based on our data, we propose that it is feasible, in Mozambique and elsewhere, to introduce a yeast such as TY13, together with normal baker's yeast in various high phytate bread types. We also suggest that adding yeast extract may further improve the nutritional value of the bread.

## AUTHOR CONTRIBUTIONS

**Ulf Svanberg:** Conceptualization (lead); funding acquisition (lead); project administration (lead); resources (equal); supervision (equal); formal analysis (lead); validation (equal); writing – review and editing (equal). **Serafina Lidia Vilanculos:** Conceptualization (equal); investigation (lead); data curation (lead); formal analysis (equal); writing – original draft (lead). **Thomas Andlid:** Conceptualization (lead); resources (equal); supervision (equal); validation (equal); writing – review and editing (equal).

## CONFLICT OF INTEREST STATEMENT

This study does not involve any conflicting interests.

## Data Availability

Research data are not shared.

## References

[fsn33754-bib-0001] Andlid, T. , Veide, J. , & Sandberg, A.‐S. (2004). Metabolism of extracellular inositol hexaphosphate (phytate) by *Saccharomyces cerevisiae* . International Journal of Food Microbiology, 97, 157–169. 15541802 10.1016/j.ijfoodmicro.2004.04.016

[fsn33754-bib-0002] Brune, M. , Rossander‐Hulten, L. , Hallberg, L. , Gleerup, A. , & Sandberg, A.‐S. (1992). Iron absorption from bread in humans inhibiting effects of cereal fiber, phytate and inositol phosphates with different numbers of phosphate groups. Journal of Nutrition, 122, 442–449. 1311753 10.1093/jn/122.3.442

[fsn33754-bib-0003] Carlsson, N.‐G. , Bergman, E.‐L. , Skoglund, E. , Hasselblad, K. , & Sandberg, A.‐S. (2001). Rapid analysis of inositol phosphates. Journal of Agricultural and Food Chemistry, 49, 1695–1701. 11308312 10.1021/jf000861r

[fsn33754-bib-0004] Dan, X. , Zhang, H. , Xi, J. , Jin, Y. , Chen, Y. , Guo, L. , Jin, Z. , & Xueming, X. (2020). Improving bread aroma using low‐temperature sourdough fermentation. Food Bioscience, 37, 100704. 10.1016/j.fbio.2020.100704

[fsn33754-bib-0005] Eduardo, M. , Svanberg, U. , & Ahrné, L. (2014). Consumers´ acceptance of composite cassava‐maize‐wheat breads using baking improvers. African Journal of Food Science, 8(7), 390–401. 10.1155/2014/479630 PMC4745537 26904634

[fsn33754-bib-0006] Egli, I. , Davidsson, L. , Juillerat, M.‐A. , Barclay, D. , & Hurrell, R. (2003). Phytic acid degradation in complementary foods using phytase naturally occurring in whole grain cereals. Journal of Food Science, 68(5), 1855–1859.

[fsn33754-bib-0007] Fredrikson, M. , Carlsson, N.‐G. , Almgren, A. , & Sandberg, A.‐S. (2002). Simultaneous and sensitive analysis of Cu, Ni, Zn, Co, Mn, and Fe in food and biological samples by ion chromatography. Journal of Agricultural and Food Chemistry, 50, 59–65. 11754542 10.1021/jf010792w

[fsn33754-bib-0008] Fretzdorff, B. , & Brummer, J. M. (1992). Reduction of phytic acid during breadmaking of whole‐meal breads. Cereal Chemistry, 69, 266–270.

[fsn33754-bib-0009] Garcia‐Estepa, R. M. , Guerra‐Hernández, E. , & Garcia‐Villanova, B. (1999). Phytic acid content in milled cereal products and breads. Food Research International, 32, 217–221.

[fsn33754-bib-0010] Haros, M. , Rosell, C. M. , & Benedito, C. (2001). Fungal phytase as a potential bread making additive. European Food Research and Technology, 213, 317–322.

[fsn33754-bib-0011] Hellström, A. , Qvirist, L. , Svanberg, U. , Veide Vilg, J. , & Andlid, T. (2015). Secretion of non‐cell‐bound phytase by the yeast *Pichia kudriavzevii* TY13. Journal of Applied Microbiology, 118(5), 1126–1136. 10.1111/jam.12767 25630750

[fsn33754-bib-0012] Hellström, A. M. , Almgren, A. , Carlsson, N.‐G. , Svanberg, U. , & Andlid, T. A. (2012). Degradation of phytate by *Pichia kudriavzevii* TY13 and *Hanseniaspora guilliermondii* TY14 in Tanzanian togwa. International Journal of Food Microbiology, 153, 73–77. 22112916 10.1016/j.ijfoodmicro.2011.10.018

[fsn33754-bib-0013] Hellström, A. M. , Vazques‐Juarez, R. , Svanberg, U. , & Andlid, T. A. (2010). Biodiversity and phytase capacity of yeasts isolated from Tanzanian togwa. International Journal of Food Microbiology, 136, 352–358. 19906458 10.1016/j.ijfoodmicro.2009.10.011

[fsn33754-bib-0014] Hinchcliffe, J. , Carlsson, N.‐G. , Jönsson, E. , Sundell, K. , & Undeland, I. (2019). Aquafeed ingredient production from herring (*Clupea harengus*) byproducts using pH‐shift processing: Effect from by‐product combinations, protein solubilization‐pH and centrifugation force. Animal Feed Science and Technology, 247, 273–284.

[fsn33754-bib-0015] Howson, S. J. , & Davis, R. P. (1983). Production of phytate hydrolyzing enzyme by some fungi. Enzyme and Microbial Technology, 5, 377–382.

[fsn33754-bib-0016] Hurrel, R. F. (2004). Phytic acid degradation as a means of improving iron absorption. International Journal for Vitamin and Nutrition Research, 74, 445–452. 15743020 10.1024/0300-9831.74.6.445

[fsn33754-bib-0017] Hurrell, R. F. , Reddy, M. B. , Burri, J. , & Cook, J. D. (2002). Phytate degradation determines the effect of industrial processing and home cooking on iron absorption from cereal‐based foods. British Journal of Nutrition, 88, 117–123. 12144715 10.1079/BJNBJN2002594

[fsn33754-bib-0018] Konietzny, U. , & Greiner, R. (2002). Molecular and catalytic properties of phytate‐degrading enzymes (phytases). International Journal of Food Science and Technology, 37, 791–812.

[fsn33754-bib-0019] Kumar, V. , Sinha, A. K. , Makkar, H. P. S. , & Becker, K. (2010). Dietary roles of phytate and phytase in human nutrition: A review. Food Chemistry, 120(4), 945–959.

[fsn33754-bib-0020] Lazarte, C. E. , Carlsson, N.‐G. , Almgren, A. , Sandberg, A.‐S. , & Granfeldt, Y. (2015). Phytate, zinc, iron and calcium content of common Bolivian food, and implications for mineral bioavailability. Journal of Food Composition and Analysis, 39, 111–119.

[fsn33754-bib-0021] Nakamura, Y. , Fukuhara, H. , & Sano, K. (2000). Secreted phytase activities of yeasts. Bioscience, Biotechnology and Biochemistry, 64, 841–844. 10830502 10.1271/bbb.64.841

[fsn33754-bib-0022] Nävert, B. , Sandström, B. , & Cederblad, A. (1985). Reduction of the phytate content of bran by leavening in bread and its effect on zinc absorption in man. British Journal of Nutrition, 53, 47–53. 2998440 10.1079/bjn19850009

[fsn33754-bib-0023] Nielsen, M. M. , Damstrup, M. L. , Dal Thomsen, A. , Rasmussen, S. K. , & Hansen, A. (2007). Phytase activity and degradation of phytic acid during rye bread making. European Food Research and Technology, 225, 173–181.

[fsn33754-bib-0024] Olstorpe, M. , Schnurer, J. , & Passoth, V. (2009). Screening of yeast strains for phytase activity. FEMS Yeast Research, 9, 478–488. 19416106 10.1111/j.1567-1364.2009.00493.x

[fsn33754-bib-0025] Porres, J. M. , Etcheverry, P. , Miller, D. D. , & Lei, X. G. (2001). Phytase and citric acid supplementation in whole‐wheat bread improves phytate‐phosphorus release and iron dialyzability. Journal of Food Science, 66(4), 614–619.

[fsn33754-bib-0026] Qvirist, L. , Carlsson, N.‐G. , & Andlid, T. (2015). Assessing phytase activity – Methods, definitions and pitfalls. Journal of Biological Methods, 2(1), 1–7.

[fsn33754-bib-0027] Qvirist, L. , Vorontsov, E. , Veide Vilg, J. , & Andlid, T. (2016). Strain improvement of *Pichia kudriavzevii* TY13 for raised phytase production and reduced phosphate repression. Microbial Biotechnology, 10(2), 341–353. 27790831 10.1111/1751-7915.12427 PMC5328827

[fsn33754-bib-0028] Raman, S. , Abdullah, N. , Boo, L. J. , Azizi, S. , & Mohamad, R. (2019). Improvement of phytase biosynthesis by new bacterial isolate *Pediococcus pentosaceus* C4/1A via continuous cultivation. Journal of Microbiology, Biotechnology and Food Sciences, 8(5), 1118–1124.

[fsn33754-bib-0029] Rosell, C. M. , Santos, E. , Sanz Penella, J. M. , & Haros, M. (2009). Wholemeal wheat bread: A comparison of different breadmaking processes and fungal phytase addition. Journal of Cereal Science, 50, 272–277.

[fsn33754-bib-0030] Sandberg, A.‐S. , & Andlid, T. (2002). Phytogenic and microbial phytases in human nutrition. International Journal of Food Science and Technology, 37, 823–833.

[fsn33754-bib-0031] Sørensen, L. S. , & Sondergaard, T. E. (2014). The effects of different yeast extracts on secondary metabolite production in Fusarium. International Journal of Food Microbiology, 170, 55–60. 24291181 10.1016/j.ijfoodmicro.2013.10.024

[fsn33754-bib-0032] Steiner, T. , Mosenthin, R. , Zimmermann, B. , Greiner, R. , & Roth, S. (2007). Distribution of phytase activity, total phosphorus and phytate phosphorus in legume seeds, cereals and cereal by‐products as influenced by harvest year and cultivar. Journal of Animal Feed Science and Technology, 133, 320–334.

[fsn33754-bib-0033] Svanberg, U. , Lorri, W. , & Sandberg, A.‐S. (1993). Lactic fermentation of non‐tannin and high‐tannin cereals: Effects on in vitro estimation of iron availability and phytate hydrolysis. Journal of Food Science, 58(2), 408–412.

[fsn33754-bib-0034] Tatala, S. , Svanberg, U. , & Mduma, B. (1998). Low dietary iron availability is a major cause of anemia: A nutrition survey in the Lindi District of Tanzania. American Journal of Clinical Nutrition, 68, 171–178. 9665111 10.1093/ajcn/68.1.171

[fsn33754-bib-0035] Türk, M. , Carlsson, N.‐G. , & Sandberg, A.‐S. (1996). Reduction in the levels of phytate during whole meal bread making; Effect of yeast and wheat phytases. Journal of Cereal Science, 23, 257–264.

[fsn33754-bib-0036] Turnlund, J. R. , King, J. C. , Keyes, W. R. , Gong, B. , & Michel, M. C. (1984). A stable isotope study of zinc absorption in young men: Effects on phytate and α‐cellulose. American Journal of Clinical Nutrition, 40, 1071–1077. 6496386 10.1093/ajcn/40.5.1071

[fsn33754-bib-0037] Vats, P. , & Banerjee, U. C. (2002). Studies on the production of phytase by a newly isolated strain of *Aspergillus niger* var teigham obtained from rotten wood‐logs. Process Biochemistry, 38, 211–217.

[fsn33754-bib-0038] Veide, J. , & Andlid, T. (2006). Improved extracellular phytase activity in *Saccharomyces cerevisiae* by modifications in the *PHO* system. International Journal of Food Microbiology, 108, 60–67. 16476497 10.1016/j.ijfoodmicro.2005.10.020

[fsn33754-bib-0039] Vieira, E. F. , Carvalho, J. , Pinto, E. , Cunha, S. , Almeida, A. A. , & Ferreira, I. (2016). Nutritive value, antioxidant activity and phenolic compounds profile of brewer's spent yeast extract. Journal of Food Composition and Analysis, 52, 44–51.

[fsn33754-bib-0040] Vilanculos, S. L. , & Svanberg, U. (2021). Degradation of phytate in composite wheat/cassava/sorghum bread by activation of intrinsic cereal phytase. African Journal of Food Science, 15(1), 1–9.

[fsn33754-bib-0041] Vilanculos, S. L. , Svanberg, U. , & Andlid, T. (2020). Degradation of phytate in composite bread by addition of phytase releasing yeast *Pichia kudriavzevii* TY13. Journal of Nutritional Science and Healthy Diet, 1(2), 30–37.

[fsn33754-bib-0042] Vilanculos, S. L. , Svanberg, U. , & Andlid, T. (2022). Phytate degradation in composite wheat/cassava/sorghum bread: Effects of preincubation of Pichia kudriavzevii TY13 and presence of yeast extract. African Journal of Food Science, 16(12), 310–318. 10.1002/fsn3.3754 PMC10804092 38268898

[fsn33754-bib-0043] Yunfei, C. , Mengmeng, L. , Jiahui, J. , Xiameng, D. , Ke, X. , Libo, J. , Yanming, Q. , & Hao, J. (2023). Advances in the application of the non‐conventional yeast *Pichia kudriavzevii* in food and biotechnology industries. Journal of Fungi, 9(2), 170. 36836285 10.3390/jof9020170 PMC9961021

[fsn33754-bib-0044] Zimmermann, M. B. , Chauoki, N. , & Hurrell, R. F. (2005). Iron deficiency due to consumption of a habitual diet low in bioavailable iron: A longitudinal cohort study in Moroccan children. American Journal of Clinical Nutrition, 81, 115–121. 15640469 10.1093/ajcn/81.1.115

